# Interactions of the cell-wall glycopolymers of lactic acid bacteria with their bacteriophages

**DOI:** 10.3389/fmicb.2014.00236

**Published:** 2014-05-22

**Authors:** Marie-Pierre Chapot-Chartier

**Affiliations:** ^1^INRA, UMR1319 MicalisJouy-en-Josas, France; ^2^AgroParisTech, UMR MicalisJouy-en-Josas, France

**Keywords:** lactic acid bacteria, bacteriophage, cell wall, phage receptor, endolysin, polysaccharide, peptidoglycan, teichoic acid

## Abstract

Lactic acid bacteria (LAB) are Gram positive bacteria widely used in the production of fermented food in particular cheese and yoghurts. Bacteriophage infections during fermentation processes have been for many years a major industrial concern and have stimulated numerous research efforts. Better understanding of the molecular mechanisms of bacteriophage interactions with their host bacteria is required for the development of efficient strategies to fight against infections. The bacterial cell wall plays key roles in these interactions. First, bacteriophages must adsorb at the bacterial surface through specific interactions with receptors that are cell wall components. At next step, phages must overcome the barrier constituted by cell wall peptidoglycan (PG) to inject DNA inside bacterial cell. Also at the end of the infection cycle, phages synthesize endolysins able to hydrolyze PG and lyse bacterial cells to release phage progeny. In the last decade, concomitant development of genomics and structural analysis of cell wall components allowed considerable advances in the knowledge of their structure and function in several model LAB. Here, we describe the present knowledge on the structure of the cell wall glycopolymers of the best characterized LAB emphasizing their structural variations and we present the available data regarding their role in bacteria-phage specific interactions at the different steps of the infection cycle.

## Introduction

**Figure 1:**
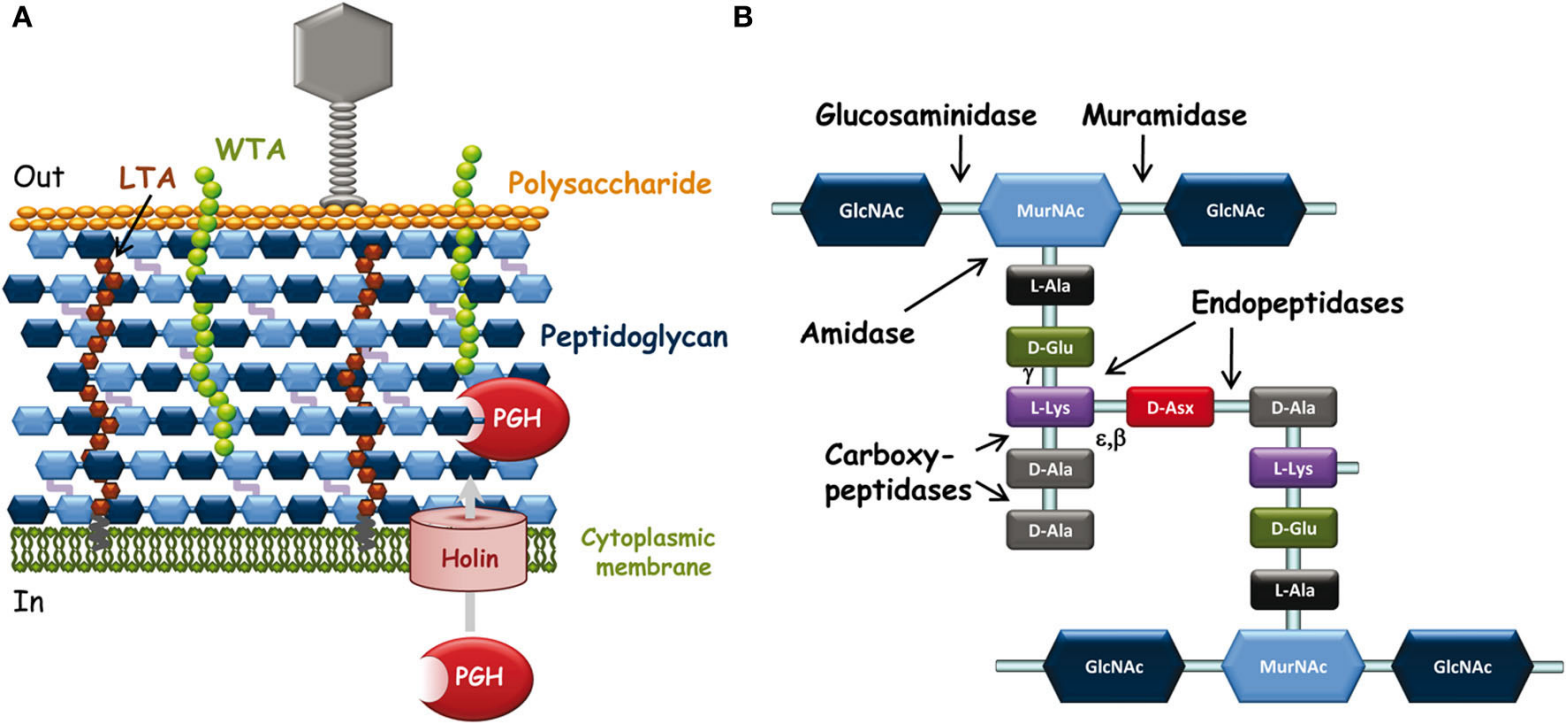
**(A)** Schematic representation of *L. lactis* cell wall and its interactions with infecting bacteriophages. Outside the bacterial cell, phages adsorb to bacteria through specific recognition of receptors (polysaccharide on this scheme) located at the bacterial surface. Inside the bacterial cell, at the end of the infection cycle, endolysin and holin encoded by the phage genome are synthesized to lyse bacteria and release phage progeny. Endolysins are PG-hydrolases that gain access to their substrate by passing through pores made by oligomerization of holins. **(B)** Schematic peptidoglycan structure. Structure with D-Asx cross-bridge (A4α-chemotype) is found in *L. lactis* and in several *Lactobacillus* species. Asx stands for Asp or Asn. The third amino acid of the stem peptide (L-Lys) may be replaced by mDAP (in *L. plantarum*) and the fifth D-Ala of the peptide stem by D-Lac in certain lactobacilli such as *L. casei*. The cross-bridge is also variable between bacterial species. The cleavage sites of the different types of PG-hydrolases are indicated by arrows. Muramidase, N-acetyl-muramidase; glucosaminidase, N-acetyl-glucosaminidase; amidase, N-acetyl-muramyl-L-Ala-amidase; endopeptidases with γ-D-Glu-L-Lys-endopeptidase or D-Ala-D-Asp-endopeptidase (specific of cross-bridge) specificity. Carboxypeptidases including D,D-carboxypeptidases and L,D-carboxypeptidases are involved in PG maturation; they were not found among endolysins.

The cell wall of Gram-positive bacteria which surrounds the cytoplasmic membrane is a complex arrangement of different biopolymers: peptidoglycan (PG), polysaccharides, teichoic acids and (glyco)proteins (Delcour et al., 1999) (Figure 1A). PG is the major component of the Gram-positive cell wall and it is made of glycan chains cross-linked through short peptide chains. It constitutes a network around the bacterial cell on which are linked covalently secondary polymers such as wall teichoic acids (WTA), polysaccharides, or LPXTG-containing proteins. Proteins can also be attached non-covalently by recognizing specific motifs of cell wall polymers or they can be organized as a layer outside the cell (S-layer). Lipoteichoic acids (LTA) anchored in the cytoplamic membrane and inserted in the cell wall contribute also to its properties and functions. The major role of the cell wall is to maintain bacterial shape and integrity. In addition, its components exposed at the bacterial surface constitute the first line of molecules to interact with abiotic or biotic environment, including eukaryotic host cells and bacteriophages.

Lactic acid bacteria (LAB) are Gram-positive bacteria widely used in food fermentations due to their ability to convert sugars into lactic acid. Lactococci and lactobacilli are used as starters in milk fermentations for the production of cheese and yogurts. They acidify milk through lactic acid production which limits food spoilage and in addition they contribute to the development of organoleptic properties including texture and flavor (Lortal and Chapot-Chartier, 2005). Bacteriophages infecting LAB constitute a real threat for dairy fermentations. Lysis of starter bacteria during their growth leads to slow or failed milk acidification, to poor quality products and finally to economic losses (Garneau and Moineau, 2011). It is expected that a better understanding of the molecular mechanisms of bacteriophage interactions with their host strain will provide new strategies to control phage infections.

During the phage infection cycle, the bacterial cell-wall components which possibly show considerable variations between species and strains are key determinants of the specific interactions of bacteriophages with their target bacteria (Samson and Moineau, 2013) (Figure 1A). First, bacteriophage particles must attach to bacteria and at this early step, cell-surface-exposed components of the bacterial wall are the likely recognized receptors (Forde and Fitzgerald, 1999). Then, phages must inject their DNA inside the bacterial cell and this step may be facilitated by PG-hydrolases (PGHs), able to locally degrade PG to make small-size holes inside the wall and allow safe passage of DNA injection device to the cytoplasmic membrane without lysing bacterial cells (Kenny et al., 2004). Finally, at the end of the infection cycle, bacteriophages make the infected cells burst to release the phage progeny; this step generally occurs by synthesis of phage-encoded PGHs, named endolysins, which recognize specifically and hydrolyze the bacterial cell wall PG (Oliveira et al., 2013).

A growing interest for the structure and function of the cell-wall glycopolymers of LAB has emerged in the past years due to their potential involvement in LAB functionality including bacterial growth and fitness, interactions with their eukaryotic host in the case of commensal and probiotic strains and sensitivity to bacteriophages. In this review, we summarize the current knowledge on the different cell wall glycopolymers including polysaccharides, teichoic acids and PG, studied mainly in four model LAB species: *Lactococcus lactis*, *Lactobacillus plantarum*, *Lactobacillus casei*, and *Lactobacillus rhamnosus.* For each component type, we present the available data regarding their role in bacteriophage infection cycle.

## Cell-wall glycopolymers as receptors of bacteriophages

The first step of bacteriophage infection is the adsorption of the phage particles to the bacterial host. This event involves recognition by phage receptor-binding proteins (RBPs) of receptors located on the target bacterial cell surface. Regarding LAB phages, until now previous studies have identified proteins as well as non-proteinaceous compounds of the cell wall such as polysaccharides or teichoic acids as phage receptors (Mahony and van Sinderen, 2012). The receptor for the C2-type group of phages infecting *L. lactis* was previously identified to be the membrane protein termed Pip (phage infection protein); adsorption of c2-phage follows a two-step process with reversible saccharide binding prior to irreversible binding to Pip protein (Geller et al., 1993; Monteville et al., 1994). In the following text, we will focus on non-proteinaceous cell wall glycopolymers identified as phage receptors.

### Cell-wall polysaccharides in LAB

The polysaccharidic components of Gram-positive bacteria surface may be divided into three groups: (i) capsular polysaccharides (CPS) that are, in most cases, covalently bound to PG and form a thick outer layer named capsule; (ii) wall polysaccharides (WPS) that may be attached to the cell wall whether or not covalently, but without forming a thick capsule; and (iii) extracellular polysaccharides (EPS) which are released into the cell environment and are not attached to the cell surface. Different polysaccharides may be produced by the same bacterium (Caliot et al., 2012), although at the experimental level it may be difficult to differentiate unambiguously the different groups.

**Figure 2:**
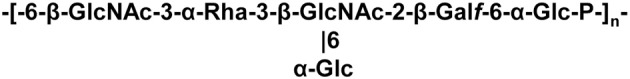
**Structure of sugar-phosphate polysaccharide pellicle of *L. lactis* MG1363**.

A WPS, which is not an EPS and capable of forming an outer layer at the bacterial surface, was discovered in *L. lactis* MG1363 (Chapot-Chartier et al., 2010). The WPS chains are composed of hexasaccharide-phosphate repeating units (Figure 2), which are distinct from other bacterial polysaccharides. Also it differs from previously characterized *L. lactis* EPS and is most probably covalently attached to the cell wall as regard the harsh acid treatment used to detach it from the bacterial cells. Atomic force microscopy (AFM) allows exploring bacterial surface architecture at the nanoscale level and was recently used to probe the surface of several LAB, including *L. lactis*, *L. plantarum*, and *L. rhamnosus* (Tripathi et al., 2012). In *L. lactis* MG1363, AFM as well as complementary transmission electron microscopy (TEM) observations show that the characterized WPS forms a compact outer layer surrounding the cell which was named pellicle (Chapot-Chartier et al., 2010). It was visualized as an electron dense layer by TEM and as a smooth layer by AFM around the cells. A derivative mutant lacking this WPS layer was obtained and was found to have a rough surface by AFM. In addition, by imaging the surface of this WPS-negative mutant with a tip functionalized with the PG-binding LysM domain, PG could be imaged as parallel cables around the bacterial cells (Andre et al., 2010). It is worth noting that a similar outer layer can be observed in a number of TEM micrographs of *L. lactis* strains of different origins, although its existence was not reported (Chapot-Chartier et al., 1994; Dabour et al., 2006). In *L. lactis* MG1363, a WPS-negative mutant makes long chains of cells which appear to have morphological defects. These observations suggest that WPS is required for normal cell division and separation. Also the WPS layer was shown to protect bacteria from phagocytosis by macrophages (Chapot-Chartier et al., 2010).

The synthesis of this WPS is encoded by a large cluster of genes in MG1363, which is conserved among *L. lactis* strains but exhibits genetic diversity that was recently analyzed in details (Mahony et al., 2013a).

Other polysaccharides associated to the cell surface were described in lactobacilli. In *L. plantarum* WSF1, four gene clusters associated with polysaccharide production are encoded in the genome (Remus et al., 2012). All these four gene clusters contribute to the overall surface polysaccharides produced by *L. plantarum*. However, in this case, the structure of the different polysaccharides has not been established until now. The surface polysaccharides were shown to influence the immunomodulatory properties of the wild-type strain probably by reducing the release or the exposure of activating molecules of the bacterial surface.

In *L. rhamnosus* GG, a long galactose-rich polysaccharide was found at the bacterial surface (Lebeer et al., 2009). This polysaccharide named EPS was detected at the bacterial surface of LGG by AFM and contributes to bacterial cell surface properties which determine adhesion and biofilm formation (Francius et al., 2009). The structure of this polysaccharide most probably corresponds to the one described earlier (Landersjo et al., 2002). The gene cluster specifying this polysaccharide in LGG exhibits differences with the clusters identified in other strains of *L. rhamnosus* in agreement with different composition of the synthesized polysaccharides (Peant et al., 2005). When the cell surface of *L. rhamnosus* was explored by AFM, it revealed a rough morphology decorated with waves (Francius et al., 2009). In contrast, a WPS-negative mutant showed a much smoother morphology suggesting that these wave-like structures reflect the production of WPS. In addition single molecule force spectroscopy with lectin-modified tips, revealed the existence of polysaccharide chains of different nature at the cell surface, polysaccharide rich in mannose or glucose having moderate extension and polysaccharide rich in galactose with much longer extensions. Deprivation of bacteria of the long galactose-rich polysaccharide results in an increased adherence and ability to form biofilm suggesting that surface adhesins such as pili structures were demasked at the bacterial surface (Lebeer et al., 2009). In addition, this polysaccharide has a protective role against host immune antimicrobial peptides (Lebeer et al., 2011).

In *L. casei* Shirota strain, two types of WPS were also described: longer, high molecular mass PS-1 and shorter low molecular mass PS-2. The gene cluster encoding PS-1 biosynthesis was identified (Yasuda et al., 2008) and PS-1 structure was previously determined (Nagaoka et al., 1990). The glycome of *L. casei* strains was compared with a lectin microarray and allowed to evidence different profiles between strains suggesting different WPS (Yasuda et al., 2011). In *L. casei* Shirota, WPS was shown also to have an immune suppressive function (Yasuda et al., 2008).

Finally, the diversity of WPS between strains of the same species was also recently observed in *Lactobacillus helveticus* strains and it was hypothesized that these different polysaccharide structures could contribute explaining the different autolytic properties observed between the studied strains (Vinogradov et al., 2013).

As a conclusion, WPS appear as omnipresent components of the cell surface of LAB and exhibit most probably high structural diversity between strains the same species.

### Cell-wall polysaccharides as bacteriophage receptors in lactococci

*L. lactis* phages are the best characterized and numerous individuals were isolated because of the wide use of *L. lactis* in dairy industrial fermentations (Garneau and Moineau, 2011). They were previously classified in 10 groups on the basis of their lytic activity on a range of *L. lactis* strains, morphology or more recently DNA-DNA hybridization and multiplex PCR. The predominant *L. lactis* phages are found in three main groups: 936, c2, and P335 species which belong to the Siphoviridae phage family, the most problematic infecting *L. lactis* and certain *Lactobacillus* species. The 936 phages are strictly lytic and thus received more specific attention because they are threatening dairy fermentations involving *L. lactis* starters (Mahony et al., 2013b). However, inside the wide 936 group, phages differ at the level of their RBPs and thereby potentially at the level of their host range (Mahony and van Sinderen, 2012).

Initial studies conducted to identify the phage receptor of 936-phages indicated that a bacterial cell-wall component differing from a protein and containing rhamnose was involved in adsorption of the phage at the bacterial surface (Valyasevi et al., 1990). Further studies using transposon random mutagenesis allowed to identify genes required for adsorption of two 936-type bacteriophages to their respective host strain. Mutations were mapped inside a gene cluster potentially involved in WPS biosynthesis (Dupont et al., 2004). Later on, the WPS of *L. lactis* MG1363 named pellicle was discovered, its structure determined and it was shown to be encoded by the corresponding gene cluster in MG1363 genome (Chapot-Chartier et al., 2010). In addition, a pellicle-negative mutant was shown to be resistant to the 936-bacteriophage sk1 strongly suggesting that this WPS consisting in hexasaccharide subunits bound through phosphodiester bonds, could be the sk1 phage receptor.

In a recent study, the gene cluster encoding WPS biosynthesis in various *L. lactis* strains was shown to contain both highly conserved regions as well as regions of high diversity, suggesting that WPS structure could be a variable character between strains (Mahony et al., 2013a). Detailed analysis of the proteins encoded in the gene cluster allowed the classification of *L. lactis* strains in three subgroups (CWPS type A, B, and C) based on the diversity regions. In parallel, a panel of 936-type phages infecting *L. lactis* was classified in different groups according to their host range and their encoded RBP sequence. Mahony et al. (2013a) revealed a correlation between the pellicle genotype of a given host strain and the host range of the tested 936-type phages. These results support the proposed role of WPS pellicle as 936-phage receptor and variations of its structure could explain the narrow host range of this type of phages. This hypothesis was very recently confirmed by the structure determination of the WPS purified from a second *L. lactis* strain with a different WPS-pellicle genotype. WPS from *L. lactis* strain 3107 was shown to be composed of pentasaccharide repeating units linked by phosphodiester bonds and thus differs from the WPS characterized in *L. lactis* MG1363. In addition, this WPS was shown to be the receptor used by several 936- phages infecting *L. lactis* 3107 (Ainsworth et al., 2014).

Remarkably, in parallel studies, the 3D-structure of the receptor binding proteins (RBPs) (also sometimes named anti-receptors) has been elucidated in several cases, including those of 936-phages p2 and bIL170 as well as P335-like phage TP901-1 (Ricagno et al., 2006; Spinelli et al., 2006a,b). These RBPs are localized at the tip of the phage tail and allow the phage to recognize specifically its receptor at the bacterial surface. The crystal structure of the protein complex connecting the RBP to the rest of the phage tail was also solved for siderophages p2 and TP901-1 (Sciara et al., 2010; Veesler et al., 2012). Recently the binding of RBP to the WPS pellicle was demonstrated in the case of the p2 RBP with the purified pellicle from its host strain MG1363 with the use of surface plasmon resonance (SPR) (Bebeacua et al., 2013). The RBP from the P335-phage TP901 which does not infect MG1363 exhibited a much lower affinity for the MG1363 pellicle. The specificity was shown to result mainly from a lower *k*_off_ value of the RBP/saccharide dissociation.

### Teichoic acids in LAB

**Figure 3:**
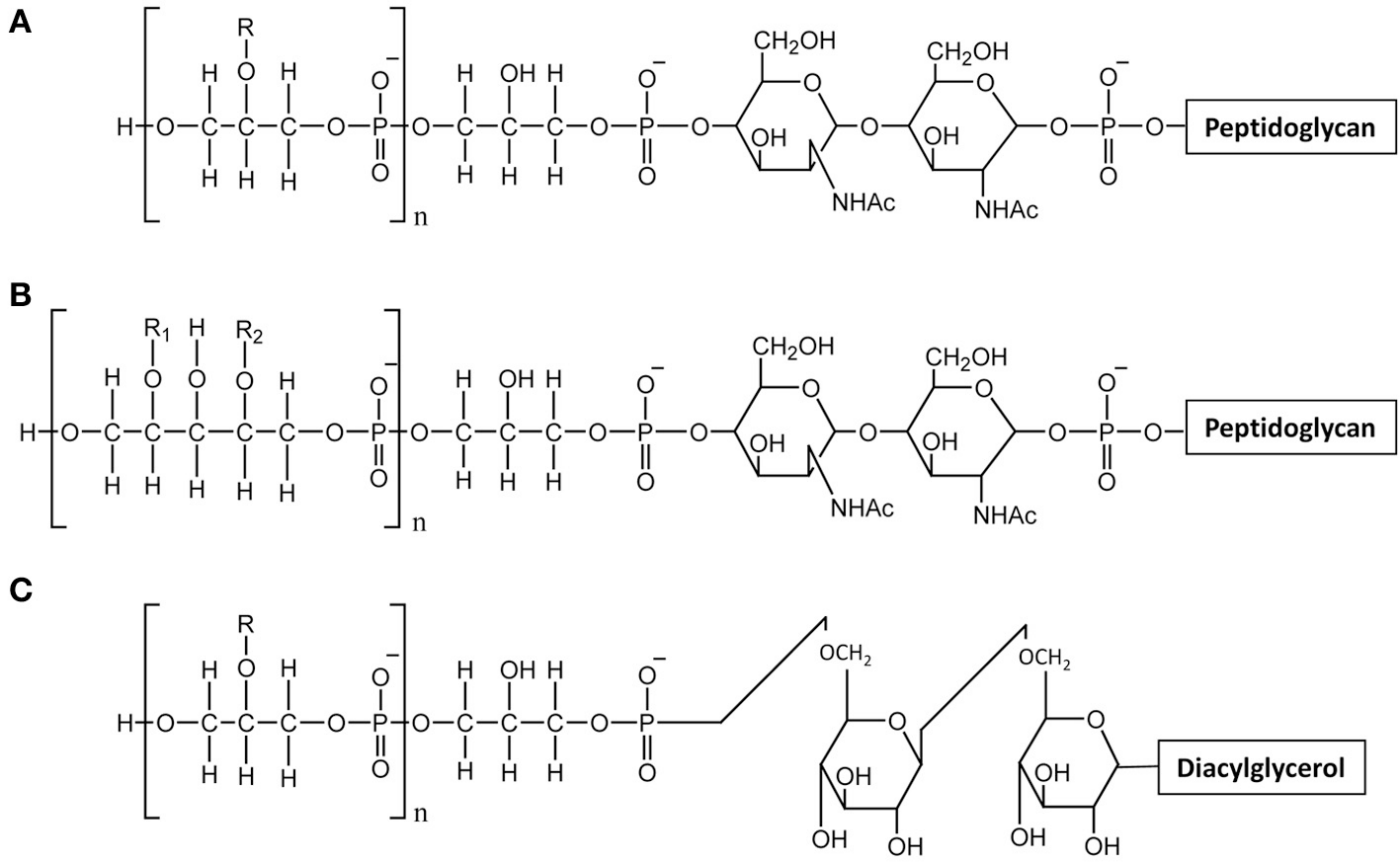
**Structure of teichoic acids. (A)** WTA with poly-glycerol-phosphate chains; **(B)** WTA with poly-ribitol-phosphate chain; **(C)** LTA with poly-glycerol-phosphate chains. R, R1, R2 indicate potential substituent groups of polyols chains (e.g., D-Ala, Glc, Gal, GlcNAc).

Teichoic acids are phosphate-rich glycopolymers that are classified into two groups: LTA anchored in the cytoplasmic membrane through a glycolipid and WTA covalently bound to PG. In certain Gram-positive bacteria such as *Bacillus subtilis*, WTA may represent up to 50% of the cell wall dry mass (D'Elia et al., 2006). WTA are quite diverse in structure but the most common ones are polymers of glycerol-phosphate (poly(Gro-P)) or ribitol-phosphate (poly(Rbo-P)) (Figure 3). With respect to LTA, the most common structure is also a poly(Gro-P) chain. It is worth noting that LTA and WTA have different biosynthetic pathways, even if they are made of similar repeating units such as Gro-P (Weidenmaier and Peschel, 2008). The glycerol or ribitol chains may be substituted with D-alanyl- or glycosyl-residues (e.g., Glc, Gal, GlcNAc) which contribute to teichoic acid functionality. In particular, D-alanyl residues provide their positive charges as counter ions of negative phosphate groups and modify the physico-chemical environment inside the cell wall and/or at the bacterial surface (Neuhaus and Baddiley, 2003).

Due to their polyanionic nature and their abundance, both WTA and LTA play multiple and varied roles in bacterial physiology. They are involved in regulation of ion homeostasis inside the cell wall, in modulating autolytic activity and in controlling cell division and morphogenesis. Also they are crucial for bacteria host interactions since their D-alanylation protect bacteria against cationic antimicrobial peptides. They also influences bacterial adhesion to abiotic surfaces and to host cells. Finally, they are recognized by the host as molecular-associated microbial patterns (MAMPS) (Brown et al., 2013; Schneewind and Missiakas, 2014).

In *L. casei or L. rhamnosus*, no WTA were detected in agreement with the absence of *tag* or *tar* biosynthesis genes, whereas in *L. lactis*, the presence of WTA remains to be further investigated. WTA have been described in *L. plantarum* strains which appear to produce either poly(Gro-P) or poly(Rbo-P) WTA. Moreover, several *L. plantarum* strains contain the genes to synthesize the two types of WTA (Bron et al., 2012). The cell surface of *L. plantarum* was also investigated by AFM combined with fluorescence microscopy with specific lectin probes (Andre et al., 2011). This approach combined with the use of specific cell-wall mutants devoid of WPS or WTA, allowed imaging the distribution of WTA at the bacterial surface. In this way it was shown that wild-type cells have a highly polarized surface morphology with smooth poles and rough lateral regions. Together with fluorescence labeling with lectin probes, AFM showed that WTA are heterogeneously distributed at the bacterial surface and absent from the surface of the poles. In addition, the complexity of *L. plantarum* surface is evidenced by the fact that PG is accessible at the surface only in absence of WPS (Beaussart et al., 2013).

The structures of both *L. rhamnosus* and *L. plantarum* LTA were confirmed to be made of a poly(Gro-P) backbone with an average of 30 and 22 repeating units of Gro-P, respectively, (Grangette et al., 2005; Claes et al., 2012b). In both cases, D-Ala was found to be the unique detectable substituent. The lipid moiety of the *L. rhamnosus* LTA reveals an average fatty acid chain of C14 (Claes et al., 2012b). In *L. lactis*, poly(Gro-P) chains contained linked D-Ala and Gal (Giaouris et al., 2008; Kramer et al., 2008).

### LTA as bacteriophage receptors in lactobacilli

A second model system where the bacteriophage receptors have been identified is the pair *Lactobacillus delbruekii* subsp. *lactis* ATCC15808 and bacteriophage LL-H. In this case, LTA were shown to be the phage receptor components (Raisanen et al., 2004). In addition, it was shown that D-Ala and α-Glc substituents of LTA affect the adsorption of LL-H phages. A high degree of D-alanylation decreased phage adsorption whereas Glc substituents were required for efficient binding (Raisanen et al., 2007). A model is proposed where the anti-receptor protein of the phage tail binds to the glucosyl- substituted glycerol of LTA, providing reversible, specificity-determining binding to the surface. Another domain of the antireceptor protein would ensure irreversible binding to the negatively charged poly-glycerol-phosphate chains (with no or low local level of D-Ala substituents) (Munsch-Alatossava and Alatossava, 2013).

## Peptidoglycan as target of bacteriophage endolysins

### Peptidoglycan structure in LAB

PG is the most abundant polymer of the Gram-positive cell wall. It is composed of glycan strands, made of alternating *N*-acetylglucosamine (GlcNAc) and *N*-acetyl-muramic acid (MurNAc), which are cross-linked by short peptide chains (Figure 1B). Although the PG basic structure is characteristic for a given bacterial species (Schleifer and Kandler, 1972), PG is in a dynamic state throughout bacterial cell life, and its structure is the result of complex biosynthetic, maturation, and degradation reactions (Typas et al., 2012).

Structural analysis of the PG-constituting muropeptides of several LAB, such as *L. lactis* (Courtin et al., 2006), *L. casei* (Regulski et al., 2012), *L. rhamnosus* (Claes et al., 2012a), and *L. plantarum* (Bernard et al., 2011a) confirmed that the first three species have a D-Ala^4^-D-Asp/Asn-L-Lys^3^ cross-bridge whereas the latter has a direct D-Ala^4^-mDAP^3^ cross-bridge. Also, PG covalent modifications were revealed, including O-acetylation of MurNAc in the four species, O-acetylation of GlcNAc in *L. plantarum*, N-de acetylation of GlcNAc in *L. lactis*, amidation of D-Asp cross-bridge in *L. lactis*, *L. casei*, and *L. rhamnosus*, and amidation of mDAP in *L. plantarum*. O-acetylation of MurNAc is known to inhibit lysozyme (Bera et al., 2005) and all the PG modifications listed above were shown to control the activity of specific endogenous bacterial PGHs (named autolysins) (Veiga et al., 2009; Bernard et al., 2011a,b).

### Hydrolysis of peptidoglycan by bacteriophage endolysins

Endolysins, encoded by phage DNA, are PGHs synthesized in phage-infected cells at the end of the multiplication cycle, and able to lyse bacteria and release phage progeny (Loessner, 2005). Endolysins usually lack a signal peptide for their export and therefore rely on the synthesis of holins which insert into the cytoplasmic membrane and make pores (Figure 1A) (Wang et al., 2000). Like bacterial PGHs, phage endolysins have a modular structure including a catalytic domain and a cell-wall binding domain (CWBD). Most often, their catalytic domain is located at the N-terminus and their CWBD at the C-terminus (Fischetti, 2008).

Generally, the catalytic domains found in endolysins belong to the same families as those encountered in bacterial PGHs (Chapot-Chartier, 2010). The different endolysins found in Siphoviridae phage genomes of *L. lactis* and different *Lactobacillus* species have been recently searched in available genome sequences (Oliveira et al., 2013) and are listed in Table 1. The catalytic domains found in these endolysins belong to five Pfam domain families which confer different hydrolytic specificities to the enzymes (Figure 1B). These domains include Amidase_2 domain (PF01510) conferring *N-acetyl-muramyl-L-Ala-amidase* activity, Glyco_hydro_25 (PF01183) conferring *N-acetyl-muramidase* activity, Phage_lysozyme domain (PF00959) conferring *N-acetyl-muramidase* activity, Amidase_5 (PF05382) conferring γ-D-Glu-L-Lys-endopeptidase activity (Regulski et al., 2013) and CHAP domain (cysteine, histidine-dependant amidohydrolase/peptidase domain) (PF05257) with both amidase and/or peptidase specificity (Frankel et al., 2011).

**Table 1:** **Domain structure of the main endolysins of Siphoviridae phages infecting *L. lactis* and *Lactobacillus* species^a^**.

**Name of the phage**	**Protein ID**	**Length (AA)**	**Catalytic domain**		**Cell wall binding domain**
			**Domain**	**Putative specificity**	
***Lactococcus lactis***					
Phage SL4	ACU46783.1	234	Amidase_2 (PF01510)	Amidase	No
Phage CB13	ACU46835.1	234			
Phage P008	YP_762533.1	233			
Phage Q54	YP_762603.1	256			
Phage bIBB29	YP_002004009.1	233			
Phage bIL170	NP_047135.1	233			
Phage P087	YP_002875753.1	237			
Phage jj50	YP_764334.1	253	Amidase_2	Amidase	No
Phage 712	YP_764281.1	258			
Phage sk1	NP_044966.1	246			
Phage r1t	NP_695077.1	270	Amidase_2	Amidase	No
Phage 949	YP_004306215.1	343	Amidase_2	Amidase	Lc-LysBD^b^
Prophage bIL285	NP_076634.1	259	Amidase_5 (PF05382)	γ-D-Glu-L-Lys- Endopeptidase	PG_binding_3 (PF09374)
Prophage bIL286	NP_076695.1	259			
Prophage bIL309	NP_076751.1	259			
Phage BK5-T	NP_116519.1	259			
Phage bIL67	NP_042321.2	226	Phage_lysozyme (PF00959)	Muramidase	No
Phage c2	NP_043551.1	226			
Phage 4268	NP_839940.1	305	Glyco_hydro_25 (PF01183)	Muramidase	No
Phage phiLC3	NP_996722.1	429	Glyco_hydro_25		2 × LysM (PF01476)
Phage TP901-1	NP_112716.1	429			
Phage Tuc2009	NP_108734.1	428			
Phage ul36	NP_663692.1	429			
Phage P335	ABI54253.1	432			
Phage 1358	ADD25719.1	233	CHAP (PF05257)	Amidase or Endopeptidase	SH3_5 (PF08460)
***Lactobacillus casei***					
Prophage Lc-Lys Phage A2	YP_001987071.1 NP_680500.1	350	Amidase_2	Amidase	Lc-LysBD^b^
Prophage Lc-Lys2	YP_001986946	324	Amidase_5	γ-D-Glu-L-Lys-Endopeptidase	Lc-LysBD^b^
Phage phiAT3	YP_025045.1	393	Glyco_hydro_25	Muramidase	SH3_5, LysM
***Lactobacillus rhamnosus***					
Phage LC-Nu	YP_358779.1	432	Glyco_hydro_25	Muramidase	2 × LysM
Phage Lrm1	YP_002117687.1				
***Lactobacillus gasseri***					
Prophage KC5a	YP_529896.1	246	Glyco_hydro_25	Muramidase	No
Phage phiadh	NP_050170.1	317	Glyco_hydro_25	Muramidase	SH3_5
***Lactobacillus delbruekii* subsp. *lactis***					
Phage LL-H	YP_001285906.1	298	Glyco_hydro_25	Muramidase	No
***Lactobacillus delbruekii* subsp. *bulgaricus***					
Phage c5	ACA63343.1	301	Glyco_hydro_25	Muramidase	SH3_5
***Lactobacillus plantarum***					
Phage LP65	YP_164723.1	464	Glyco_hydro_25	Muramidase	No
Phage phiJL-1	YP_223905.1	398	Glyco_hydro_25	Muramidase	SH3_5
Phage Sha1	ADW01314.1	390	Glyco_hydro_25	Muramidase	SH3_5 LysM
Phage phig1e	YP_003084340.1	442	Glyco_hydro_25	Muramidase	SH3_5 LysM
***Lactobacillus johnsonii***					
Prophage Lj928	NP_958555.1	315	Glyco_hydro_25	Muramidase	SH3_5

a Data extracted from Oliveira et al. (2013).

b Lc-LysBD was characterized in Regulski et al. (2013).

Interestingly, tail-associated lysins were also found in certain bacteriophages such as Tuc2009 and TP901-1. The tail fiber of these phages is composed of a trimer of Tal proteins which contain a PG-hydrolase domain of the M23-peptidase family (PF01551). This domain is protruding from the large host-recognizing baseplate structure of each of these phages (Kenny et al., 2004) and is most likely involved in PG digestion required for phage DNA injection inside the cytoplasm thus facilitating infection especially when PG is highly cross-linked. The hydrolytic specificity of the Tal PGH was shown to be a D-Ala-D-Asp/Asn endopeptidase allowing hydrolysis of PG peptide cross-bridges (Figure 1B), potentially making holes in the PG network (Stockdale et al., 2013).

### Peptidoglycan as ligand of bacteriophage endolysin CWBDs

The CWBD of bacteriophage endolysins is thought to maintain the proteins tethered to the cell wall after bacterial lysis. This will allow preventing further attack and lysis of adjacent bacterial cells that represent potential hosts for the new phage particles released upon lysis thus ensuring phage propagation. Very often endolysin CWBDs bind cell wall with high affinity and high specificity. Therefore, they were proposed for biotechnological applications such as identification of bacteria by specific staining (Schmelcher et al., 2010) or, after fusion with a protein of interest, for displaying this protein at the bacterial surface with potential applications such as vaccine or biocatalyst development (Lee et al., 2003; Visweswaran et al., 2014).

*Lactococcus* and *Lactobacillus* endolysins exhibit high diversity in their CWBD (Oliveira et al., 2013) and a number of them contain cell-wall binding modules commonly found in bacterial PGHs such as LysM or SH3b. However, a large number of endolysins do not display any sequence similarity in their C-terminal part with other known proteins and this C-terminal part could contain uncharacterized cell-wall binding modules.

The LysM module (PF01476) consists of a sequence motif of about 40 residues, which is widespread in eukaryotic and prokaryotic proteins, and often present as several repeats constituting a LysM-domain. It was found in several LAB PGHs such as the *L. lactis* major autolysin AcmA. LysM modules were shown to bind glycan chains of PG, involving most probably GlcNAc (Steen et al., 2003; Frankel and Schneewind, 2012).

The SH3 domain initially known in eukaryotes and virus was later on identified in bacterial PGHs. SH3 bacterial domains named SH3b (including different subfamilies SH3_3, SH3_4, and SH3_5) were reported to bind PG; however contradictory results were published regarding the exact recognized motif. It was concluded that the SH3-containing domain of ALE-1, an homolog of lysostaphin produced by *Staphylococcus simulans*, binds PG and that the length of the interpeptide cross-bridge and its amino acid composition have a major impact on the binding (Lu et al., 2006). Another study revealed that the C-terminal domain of lysostaphin which contain SH3_5 domain direct the enzyme to cross-linked PG (Grundling and Schneewind, 2006). In contrast, single molecule AFM experiments with tips functionalized with Acm2, the *L. plantarum* major autolyin containing five SH3_5 domains, concluded that SH3b domains rather bind PG glycan chains and involved GlcNAc (Beaussart et al., 2013).

Recently a CWBD, not described before, was characterized in the C-terminal part of prophage endolysins (Lc-Lys and Lc-Lys2) found in the complete genome sequence of *L. casei* BL23 (Regulski et al., 2013). This domain did not exhibit sequence identity with any known CWBD. It was demonstrated to bind PG and to be highly specific for amidated D-Asp cross-bridge present in *L. casei* PG (Figure 1B). It does not bind PG with another type of crossbridge such as L-Ala-L-Ala/L-Ser or even PG with non-amidated D-Asp cross-bridge. This domain (named Lc-LysBD) is also present in endolysins of other *L. casei* phages A2 and PL-1 as well as in *L. lactis* phage 949 endolysin (Table 1).

Another PG-binding domain (PG_binding_3 (PF09374)) is found in the C-terminal part of several endolysins listed in Table 1. However, the exact motif recognized by this domain is unknown.

## Conclusions-perspectives

The cell wall of LAB has received increased attention in the recent past years. Advances in structural studies of the cell wall and its components allow now the investigation of the molecular mechanisms of the interactions between bacteriophages and their host bacteria at several steps of the infection cycle. Further studies will aim at elucidating the inter-strain structural diversity of cell-wall polymers that are phage receptors at the bacterial surface, which could explain the narrow host range of certain *L. lactis* phages. Furthermore, the 3D-structures of several RBPs are available and the molecular determinants of the specificity of the binding of RBPs to the polysaccharide receptors can now be investigated. At the applied level, further knowledge will allow rational selection of LAB strains taking into account their WPS-types to design starters resistant to certain groups of bacteriophages with known RBPs or for strain rotation to prevent phage attack. Also, as already proposed previously with the use of camelid nanobodies raised against the purified baseplate complex (Desmyter et al., 2013), strategies based on the inhibition of the binding of RBP to their receptors may be considered at the molecular level on the basis of the 3D-structures of RBPs. In another field of applications, it is expected that new CWBDs could be discovered in phage-encoded endolysins and their ligands in the cell wall characterized. This improved knowledge will open new perspectives to construct tools to display proteins of interest at the bacterial surface of LAB for biotechnological applications.

### Conflict of interest statement

The author declares that the research was conducted in the absence of any commercial or financial relationships that could be construed as a potential conflict of interest.
